# Cortisol coregulation in fish

**DOI:** 10.1038/srep30334

**Published:** 2016-07-26

**Authors:** Ines Fürtbauer, Michael Heistermann

**Affiliations:** 1 Department of Biosciences, College of Science, Swansea University, SA2 8PP Swansea, UK; 2 Endocrinology Laboratory, German Primate Center, Kellnerweg 4, 37077 Göttingen, Germany

## Abstract

Cortisol coregulation, which is the up- or down-regulation of partners’ physiological stress responses, has been described for individuals with strong attachment bonds, e.g. parents and their children, and romantic relationship partners. Research into moderating effects on cortisol coregulation suggests stronger covariation among distressed partners. Whether cortisol coregulation is unique to humans or can also be found in other species that share universal features of the vertebrate stress response remains unexplored. Using a repeated measures approach and non-invasive waterborne hormone analysis, we test the hypothesis that dyads of three-spined stickleback fish (*Gasterosteus aculeatus*) coregulate their cortisol levels in shared environments. Dyadic cortisol levels were unrelated when cohabiting (home tank), but significantly covaried when sharing a more stressful (as indicated by higher cortisol levels) environment (open field). Time-lag analysis further revealed that open field cortisol levels were predicted by partner’s cortisol levels prior to the shared experience. To our knowledge, this study provides the first evidence for coregulatory processes on cortisol responses in a non-human animal that lacks strong bonds and social attachment relationships, suggesting a shared evolutionary origin of cortisol coregulation in vertebrates. From an adaptive perspective, cortisol coregulation may serve to reduce risk in challenging, potentially threatening situations.

Social relationships can have a powerful impact on both mental and physical health^1^,^2^,^3^,^4^,^5^,^6^,^7^. The availability and perception of social support, in particular, have been shown to play an important role in the recovery from stressful experiences, a phenomenon commonly described as ‘social buffering’^1^,^8^,^9^. More recently, however, research on human subjects has discovered that individuals with strong attachment bonds (typically family members) can influence each other’s physiological states or, in other words, they ‘coregulate’^10^. Cortisol coregulation, in contrast to social buffering which, by definition, is unidirectional, describes the phenomenon of a bidirectional interdependence of social partners’ hypothalamic–pituitary–adrenal (HPA) axis activity, i.e. an up- or down-regulation of physiological stress (cortisol) levels^11^,^12^,^13^. Cortisol covariation has been documented between parents and their children^11^,^14^,^15^,^16^,^17^ and in cohabiting intimate couples^11^,^12^,^13^,^18^.

Generally, cortisol coregulation is thought to be a “physiological manifestation of shared emotional and behavioral experiences”^19^. Investigations into coregulatory processes of HPA axis activity revealed, for example, that physical closeness–when individuals share an environment or activities–leads to stronger cortisol covariation^12^,^13^,^20^. Furthermore, distress situations, such as domestic violence, appear to be related to stronger cortisol covariation between pair partners, and mothers and their infants^11^,^21^.

Cortisol coregulation between parents and children is thought to be of adaptive value since children’s self-regulatory capacities are not yet independent, and thus, might be critical for child development^10^,^22^. Although cortisol coregulation in adults is unlikely to be a by-product of child-parent relationships, its adaptive function is unclear^10^. To date, studies into the interplay of adult partners’ HPA axis activity (and other measures of physiological arousal) have involved human individuals with strong attachment bonds and have focussed on the potential implications for relationship functioning, presuming that coregulation becomes activated due to threats to the bond. However, according to social baseline theory, physiological coregulation may represent the most efficient way to save energy and reduce risk in threatening contexts, in a more general sense^23^. Moreover, the susceptibility of the HPA axis to social modulation is well documented in non-human animals^2^,^8^,^9^,^24^. Thus, cortisol coregulation may represent a general mechanism that is not unique to human attachment relationships and also exist in species that lack human-like bonds. However, to the best of our knowledge, the phenomenon of cortisol coregulation has not yet been studied in non-human animals, and it remains unclear whether cortisol coregulation is unique to humans, or an ancestral trait of vertebrates.

In the present study, we test the cortisol coregulation hypothesis in a basal vertebrate, a freshwater teleost fish, the three-spined stickleback (*Gasterosteus aculeatus*), using an experimental approach with non-invasive waterborne cortisol analysis and a repeated measures design^25^,^26^. Sticklebacks provide ideal models for studying the potential interdependence of social companions’ cortisol responses. First, they are gregarious and respond to shoal partners. For example, in potentially threatening situations, fishes’ time to react to conspecifics decreases and shoal synchrony increases^27^,^28^. Second, the fish hypothalamic–pituitary–interrenal (HPI) axis (similar to the mammalian HPA axis^29^,^30^) responds to a stressor within minutes^30^. Third, sticklebacks (and other fish) are susceptible to social mediation of HPI axis activity^31^. Therefore, inspired by and analogous to human research, we consider different processes potentially affecting cortisol coregulation in sticklebacks. We sample our fish in three different environmental contexts: (i) when cohabiting, which is analogous to the human “at home together” scenario^12^,^13^,^20^ (Fig. 1A), (ii) when in a shared, potentially stressful open field environment, which is analogous to the human “distress situation”^11^,^21^ (Fig. 1B), and (iii) when in an unshared environment, which is analogous to the human “at work” scenario^12^,^13^,^17^ (Fig. 1C). In accordance with the cortisol coregulation hypothesis, we first predict that fish dyads’ cortisol levels correlate in shared but not in unshared environments, and second, we expect stronger dyadic covariation in a potentially threatening compared to a non-threatening environment.

## Results

Fish exhibited significantly higher waterborne cortisol concentrations in an open field compared to a cohabiting context (estimate ± se: 0.26 ± 0.06, t = 4.58, p < 0.001; Fig. 2; full vs. null model: χ^2^ = 53.2, df = 6, p < 0.001, n = 263; Table S1), indicating that the open field was perceived as a stressor. Cortisol levels were unrelated to fish sex and weight across contexts (sex: estimate ± se: −0.15 ± 0.08, t = −1.85, p = 0.077; weight: estimate ± se: 0.19 ± 0.10, t = 1.93 p = 0.065; Table S1), and were lower in the afternoon than in the morning (estimate ± se: −0.22 ± 0.06, t = −3.77, p < 0.001; Table S1).

In accordance with our first prediction, we found a significant interaction between partner cortisol and the shared open field (estimate ± se: 0.55 ± 0.14, t = 3.99, p < 0.001; Fig. 3; Table S1) but not the unshared open field context (estimate ± se: 0.08 ± 0.15, t = 0.55, p = 0.582; Fig. 3; Table S1), indicating that the dyadic covariation of cortisol levels differed across environmental contexts. Further analysis revealed that cortisol levels of dyad partners were unrelated when cohabiting in their home tank (estimate ± se: − 0.05 ± 0.09, t = − 0.60, p = 0.550; Table S1) and in an unshared open field (estimate ± se: − 0.04 ± 0.11, t = − 0.37, p = 0.716; Table S1). Conversely, dyad partners’ cortisol levels were significantly positively correlated when sharing an open field (estimate ± se: − 0.67 ± 0.12, t = 5.54, p < 0.001; Fig. 4A; Table S1). This indicates dyadic coregulation of HPI axis activity in a shared, more stressful environment, which is in line with our second prediction. Time-lag analysis revealed that partners’ cohabiting cortisol levels (‘baseline’; prior to the open field test) predicted focal fish cortisol responses in the shared open field environment (estimate ± se: 0.35 ± 0.14, t = 2.56, p = 0.015; Fig. 4B; Table S1). In other words, individuals’ cortisol levels were higher following the shared open field situation when their dyad partner exhibited higher baseline cortisol.

## Discussion

Research into human cortisol coregulation (or ‘linkage’) has increased in recent years but many questions still remain unanswered^18^,^22^. This lack of knowledge may be at least partly attributed to the focus on individuals with strong bonds, for example romantic relationship partners, and parents and their children^18^,^22^. The findings of this study represent, to the best of our knowledge, the first empirical evidence for comparable coregulatory processes on cortisol responses in a non-human animal species that lacks strong social bonds and attachment relationships. Using fish as an animal model of cortisol coregulation could help to obtain valuable information regarding its mechanisms and functions in humans. Indeed, fish models are becoming increasingly popular due to the intriguing similarities between fish and human socio-endocrinological patterns^32^.

In humans, cortisol levels in socially attached individuals appear to be only associated when individuals are in a shared environment, but not when in an unshared environment^12^. Furthermore, stronger cortisol linkage is found in stressful contexts^11^,^21^. Similarly, cortisol levels in our stickleback dyads were only associated when fish were in a shared, more stressful environment (as indicated by higher cortisol levels) but not in an unshared environment or when cohabiting. This suggests that dyads’ HPI axis activities do not become entrained by cohabiting but that coregulatory processes become activated under stress, and furthermore, can operate at a very fast time scale (i.e. minutes), which is in line with other examples of rapid, socially modulated endocrine plasticity^2^,^33^.

Our findings therefore raise the question: what mechanisms underlie cortisol linkage in a perceived threatening situation? Research into social modulation of mammalian stress responses has shown that, for example, social buffering effects, i.e. the down-regulation of stress responses through the presence/behaviour of a social partner, are mediated by tactile, chemical, and/or visual cues^34^,^35^,^36^,^37^. In fish, both chemical and visual cues are of major importance for inter-individual communication^38^, and are likely to also underlie the communication of partners’ stress statuses^39^. Given the bidirectional nature of cortisol coregulation, it is possible that different chemicals mediate up- and down-regulation of cortisol responses. For example, in pacus (*Piaractus mesopotamicus*), behavioural responses to different predator conditions, i.e. alert (predator condition) and attraction (non-predator condition) appear to be mediated by at least two different chemicals, or different proportions of these chemicals^40^. Furthermore, recent work on zebrafish (*Danio rerio*) revealed that the chemical communication of predation risk is independent of a surge in cortisol levels in ‘donor’ fish but can still lead to changes in HPI axis activity^41^. Visual cues, such as changes in or the occurrence of certain behaviours in response to unpredictable, potentially threatening environments may also trigger coregulatory physiological processes. If behavioural cues indeed are crucial in the aforementioned context, future research should assess whether familiarity with a social partner is necessary for coregulatory processes on cortisol responses to occur. Partner familiarity has been shown to be a major moderating factor on social buffering effects in birds and mammals^42^,^43^,^44^ and, in fish, can affect behavioural responses and social interactions in novel situations^45^,^46^.

Another interesting finding was revealed by our time lag analysis, showing that when paired with a high-stress partner (i.e. an individual with high ‘baseline’ cortisol levels), fish had higher cortisol levels following the shared open field experience (Fig. 4B) than when paired with a low-stress companion. Generally, our finding suggests that partner’s baseline stress reactivity is an important modulator of coregulatory processes of HPI axis activity in stickleback fish and potentially also of HPA axis activity in humans. Future human studies may therefore benefit from investigating whether partners’ baseline cortisol levels can be used to predict up- or downregulation of cortisol levels in stressful contexts. This could help to further illuminate if and when cortisol coregulation is “good” or “bad”, an issue still under debate^18^.

On a functional level, physiological coregulation has been proposed to reduce energy expenditure and risk^23^. In fact, risk dilution is one of the main reasons for group cohesion and behavioural synchrony in social animals, both of which often increase in threatening contexts^47^,^48^. In fish, shoaling (i.e. the tendency to stick with others) and swimming speed become more synchronous under a perceived threat^27^. Moreover, research into ‘stress coping styles’, has revealed consistent links between physiological and behavioural stress responses, i.e. reactive (high physiological stress, immobility) versus proactive styles (low physiological stress, high locomotor activity) across and within individuals^26^,^49^. Stickleback fish, for instance, are shyer on days they exhibit higher cortisol levels^26^. Thus, we hypothesize that cortisol coregulation may underlie synchronized collective behavioural responses, and, ultimately may serve to enhance risk dilution in potentially threatening situations.

Overall, the present study is an important contribution and advances our understanding of cortisol coregulation, a phenomenon which to date was thought to be unique to human attachment relationships. The interplay of fishes’ physiological stress responses in the absence of strong social bonds hints at a shared evolutionary origin of cortisol coregulation in vertebrates and suggests that it is not simply a by-product of partners’ shared environments. Cortisol coregulation potentially confers important adaptive value across vertebrates, which future studies should look into.

## Methods

### Subjects and experimental setup

**Figure 1:**
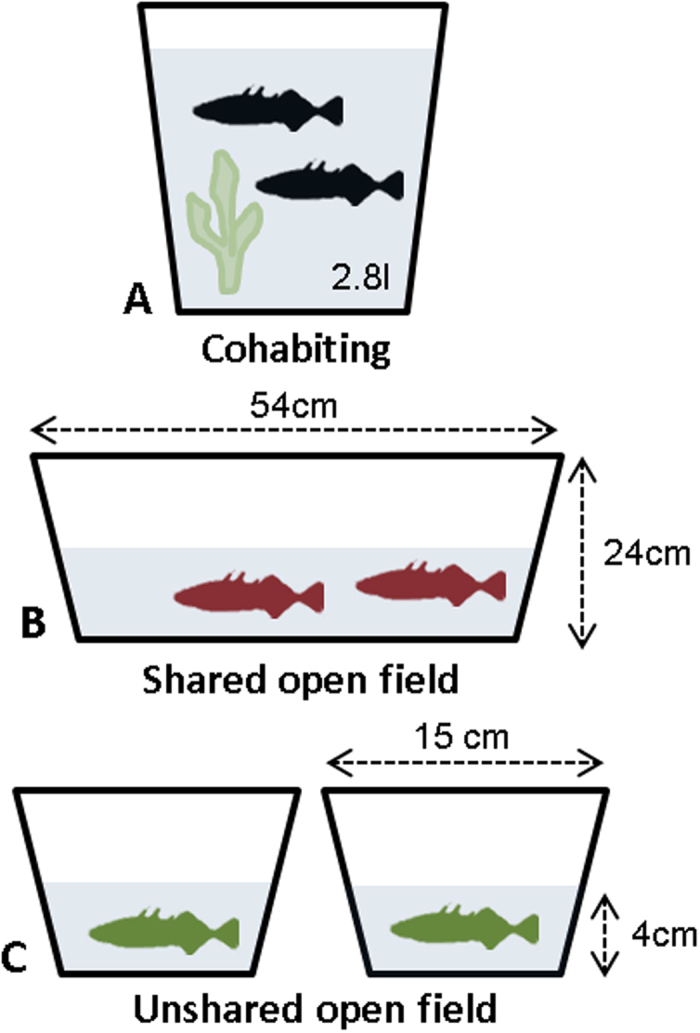
Experimental setup for investigating cortisol coregulation in dyads of three-spined stickleback fish. Fish dyads were sampled in each of three environmental contexts, i.e. when cohabiting (**A**) and after experiencing a shared (**B**) or unshared (**C**) open field environment. Note that test tank sizes (54 × 15 × 24 cm) and water levels (4 cm) were identical in both open field conditions (**B**,**C**), and that fish had no visual contact in the unshared open field context (**C**). Sampling order was the same for all individuals and for each context, all fish were tested within the same day and the two individuals of a dyad were sampled at the same time. The fish were kept and tested with their partner throughout the study and no re-grouping of dyads took place.

**Figure 2:**
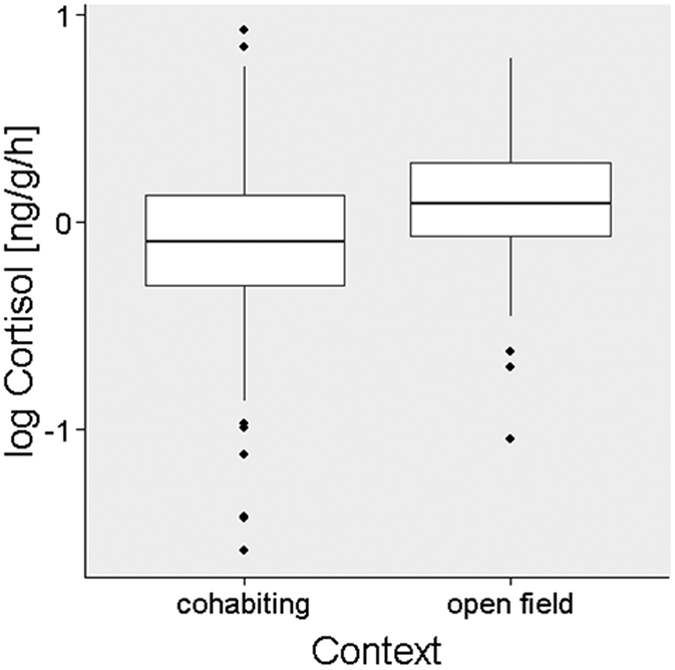
Waterborne cortisol concentration when cohabiting and experiencing an open field environment. Fishes’ cortisol levels were significantly higher in an open field context compared to a cohabiting context.

**Figure 3:**
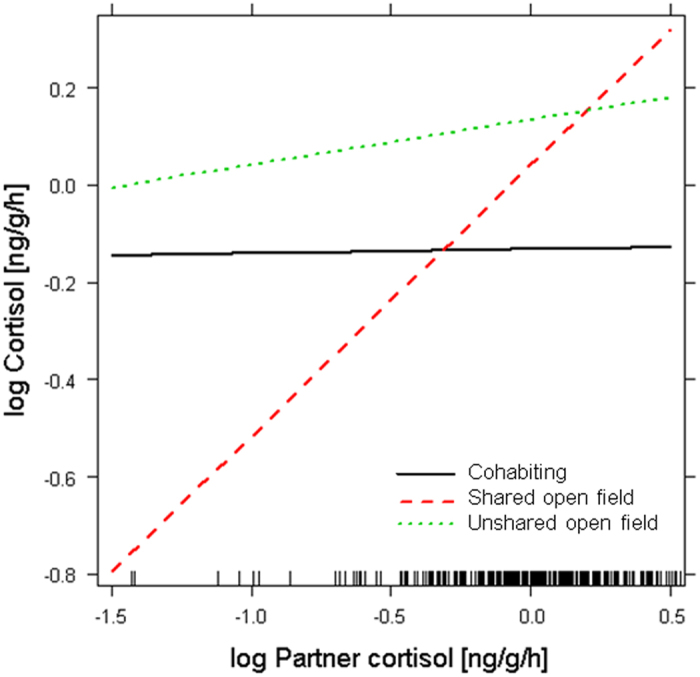
Partner fish cortisol predicting focal fish cortisol, as moderated by environmental context. Dyads’ cortisol levels were significantly positively correlated in a shared open field (red dashed line) but not in a shared home tank (black solid line) or an unshared open field (green dotted line). See text for details.

**Figure 4:**
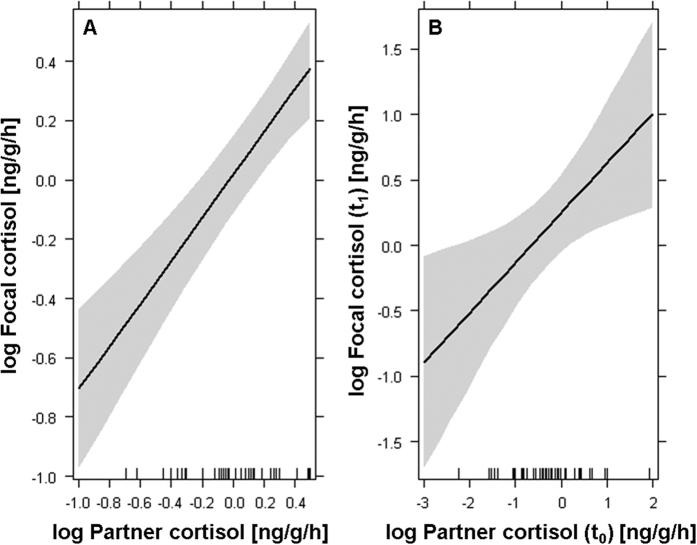
Covariation in dyadic cortisol levels (**A**) in a shared open field environment and (**B**) between focal fish open field cortisol (t_1_) and partners’ baseline cortisol prior to the open field test situation (t_0_). Effects shown are predictions from LMMs. The shaded areas indicate upper and lower 95% confidence limits.

N = 22 fish, sourced from a wild population on Swansea University Campus, were initially housed together in a large tank (30 × 29 × 122 cm). Fish were fed daily with defrosted bloodworms (*Chironomus sp.*). Throughout the study, the fish were kept under non-breeding environmental conditions (16 °C/8L:16D). Four days prior to the experiments, 11 fish dyads (paired randomly with respect to sex; mean weight ± SD: 1.63 ± 0.38 g) were transferred to individual 2.8l gravel-lined aerated tanks (Fig. 1A). The fish were kept and tested with their original partner throughout the study and no re-grouping took place. Fish were tagged with Visible Implant Elastomer (VIE) tags (Northwest Marine Technology, Inc., 2014; www.nmt.us/products/vie/vie.shtml), a harmless method for individual identification of small fish^25^. Each fish was sampled repeatedly in three different contexts: (1) cohabiting (n = 6 samples per fish; Fig. 1A), (2) shared open field (n = 2 samples per fish; Fig. 1B) and (3) unshared open field (n = 4 samples per fish; Fig. 1C). Samples were collected over a 10-day period; sampling order was the same for all individuals and for each condition, all fish were tested within the same day and the two individuals of the dyad were sampled at the same time. The open field test tanks were identical in the shared and unshared contexts (54 × 15 × 24 cm) and were filled with water up to 4 cm, and the water (same source as for home tanks and hormone collection) was changed between each trial to decrease potential olfactory cues (Fig. 1). The fish were exposed to the shared/unshared open field for 30 minutes. All procedures described were carried out in accordance with the guidelines for the use of animals approved by the Association for the Study of Animal Behaviour and were approved by Swansea University’s Ethics Committee (IP-1213-3).

### Hormone collection, extraction, and analysis

A total of n = 263 waterborne hormone samples were collected and analysed for cortisol, following published procedures^25^,^26^. Briefly, either straight out of the home tank or after experiencing a shared/unshared open field environment, fish were placed individually in 150 ml glass beakers filled with 50 ml water (same source as used for home tanks) for one hour. All subjects were exposed to beaker confinement for habituation twice prior to experiments^26^. Net-filtered water samples were collected in 60 ml polypropylene bottles and stored at −18 °C until extraction. Steroids were extracted using solid phase extraction procedures and using Sep-Pak Plus C18 solid phase extraction Cartridges (Waters #WAT020515), and a Visiprep 12-port vacuum manifold connected to a KNF Laboport vacuum pump. Steroids were eluted from the cartridges with 5 ml methanol, collected in a glass tube and evaporated under nitrogen at 45 °C. Steroids were redissolved in 350 μl assay buffer, and analysed using an enzyme immunoassay for immunoreactive cortisol^50^. All samples were run in duplicate, and samples with a c.v. >7% between duplicates were re-measured. Sensitivity of the assay at 90% binding was 0.5 pg. Intra- and inter-assay c.v., calculated from replicate determinations of high and low-value quality controls, were 7.4% (n = 16) and 7.2% (n = 10) (high) and 8.9 (n = 16) and 12.7% (n = 10) (low).

### Statistical analyses

All data were analysed using Linear Mixed Models (LMM^51^) fitted in R (R Development Core Team: www.r-project.org), using the package *lme4*^52^. Cortisol data were log-transformed to normalise model residuals. In all models, fish sex, weight, and sampling time (A.M. versus P.M.) were controlled for. To allow for individual- and dyad-specific differences, random intercepts were fitted for “Individual” and “Dyad”. The significance of the full models as compared to the null models was established using likelihood ratio tests (R function *anova*). Co-linearity of fixed effects was controlled for by calculating Variance Inflation Factors^53^ for standard linear models excluding the random effects. Model diagnostics were performed using graphical procedures (Q-Q plot and standardized residuals vs. fitted values).

First, in order to test whether the open field environment was perceived as a stressor, we tested for overall differences in cortisol concentrations between cohabiting and open field contexts (model 1; Table S1). The model was fitted with a random slope for context. Second, to simultaneously test for a correlation between partners’ cortisol levels and a potential moderating effect of environmental context (cohabiting, shared and unshared open field), we fitted a LMM with cortisol as response and a two-way interaction between partner cortisol and context (cohabiting was used as reference variable) (model 2, Table S1). The model was fitted with a random slope for context. Third, since significant interaction terms make the interpretation of main effects of the interacting predictors unreliable^54^, we ran separate models for each environmental context (models 3–5; Table S1). Fourth, to further assess coregulatory influences in a shared open field context, we ran a time-lag analysis, and modelled open field cortisol levels (t_1_) as a function of partners’ ‘baseline’ cortisol levels prior to the occasion (t_0_) (model 6; Table S1). The time interval between t_0_ and t_1_ and open field hormone collection was the same for all individuals (1.5 hours between the start of each of the two hormone collections). For all analyses, the level of significance was set at P < 0.05.

## Additional Information

**How to cite this article**: Fürtbauer, I. and Heistermann, M. Cortisol coregulation in fish. *Sci. Rep.* **6**, 30334; doi: 10.1038/srep30334 (2016).

## Supplementary Material

Supplementary Information
