# Supplementary material for: Cortisol coregulation in fish
Source: Sci Rep. 2016 Jul 26;6:30334. doi: 10.1038/srep30334 (PMC4960609; doi:10.1038/srep30334)
Supplement: Supplementary Information [file srep30334-s1.pdf]

Cortisol coregulation in fish

Ines Fürtbauer & Michael Heistermann

Supplementary Material

Table S1: Summary of Linear Mixed Models investigating cortisol coregulation in three-spined stickleback fish.

Model	Response variable	Predictor variable	estimate±se	t value	p-value
Model 1	Cortisol	Intercept	-0.26±0.17		
n=263		Context open field	0.26±0.06	4.583	**<0.001**
		Time PM	-0.22±0.06	-3.766	**<0.001**
		Sex	-0.15±0.08	-1.853	0.077
		Weight	0.19±0.10	1.927	0.065
Model 2	Cortisol	Intercept	-0.22±0.16		
n=262		*Partner cortisol*	*0.01±0.08*	*0.110*	*0.913*
		Context Shared open field	*0.17±0.07*	*2.340*	*0.030*
		Context Unshared open field	*0.27±0.07*	*4.096*	*<0.001*
		Partner cortisol:Shared open field	0.55±0.14	3.993	**<0.001**
		Partner cortisol:Unshared open field	0.08±0.15	0.551	0.582
		Time PM	-0.18±0.06	-3.155	0.002
		Sex	-0.12±0.07	-1.584	0.123
		Weight	0.15±0.10	1.530	0.137
Model 3	Cohabiting Cortisol	Intercept	-0.30±0.23		
n=130		Partner cortisol	-0.05±0.09	-0.600	0.550
		Time PM	-0.31±0.09	-3.608	**<0.001**
		Sex	-0.23±0.11	-2.059	**0.049**
		Weight	0.26±0.14	1.834	0.075
Model 4	Shared open field Cortisol	Intercept	-0.09±0.29		
n=44		Partner cortisol	-0.67±0.12	5.541	**<0.001**
		Time PM	-0.12±0.12	-0.988	0.329
		Sex	-0.03±0.13	-0.199	0.844
		Weight	0.12±0.18	0.690	0.498
Model 5	Unhared open field Cortisol	Intercept	0.20±0.18		
n=88		Partner cortisol	-0.04±0.11	-0.366	0.716
		Time PM	0.06±0.08	0.852	0.402
		Sex	-0.04±0.09	-0.498	0.625
		Weight	0.12±0.18	-0.496	0.625
Model 6	Shared open field Cortisol	Intercept	0.004±0.28		
n=43		Partner cortisol before occasion	0.35±0.14	2.555	**0.0149**
		Time PM	-0.27±0.13	-2.111	**0.0426**
		Sex	-0.07±0.13	-0.543	0.5937
		Weight	0.18±0.18	1.050	0.3063

Factors included in significant interaction terms cannot be interpreted as independent variables (in italics and indented).
